# Loss of Lactate/Proton Monocarboxylate Transporter 4 Induces Ferroptosis via the AMPK/ACC Pathway and Inhibition of Autophagy on Human Bladder Cancer 5637 Cell Line

**DOI:** 10.1155/2023/2830306

**Published:** 2023-01-21

**Authors:** Siqi Dong, Lei Zheng, Tao Jiang

**Affiliations:** Department of Andrology, The Second Hospital of Dalian Medical University, Dalian116000, China

## Abstract

**Background:**

Ferroptosis and autophagy have an important role in the occurrence and development of cancer, and lactate in cells and microenvironment is one of the influencing factors of ferroptosis and autophagy. The lactate/proton monocarboxylate transporter 4 (MCT4), which is expressed in the cell membrane, regulates the transport of intracellular lactic acid and lactate. The knockout of MCT4 can affect intracellular and extracellular lactic acid levels, thereby affecting the growth, proliferation, and metastasis of tumor cells via regulation of the oxidative stress in cells. However, whether MCT4 affects ferroptosis and autophagy in bladder cancer cells remains unclear.

**Methods:**

Colony formation assay and bladder cancer xenograft animal model were used to assess the effect of MCT4 on the growth in 5637 cells. Reactive oxygen species (ROS) assay, lipid ROS assay, lipid peroxidation assay (MDA), and transmission electron microscopy were performed to assess the level of lipid peroxidation in 5637 cells. RNA-sequence, RT-PCR, and Western Blot were used to analyze the mechanism of MCT4 of ferroptosis and autophagy. AdPlus-mCherry-GFP-LC3B reporter system was used to detect the effect of MCT4 on autophagy in 5637 cells, and the effect of knockdown of MCT4 on apoptosis was analyzed by flow cytometry.

**Results:**

The mRNA level of MCT4 was significantly upregulated in patients with bladder cancer, which was associated with a poor prognosis. *In vivo* and *in vitro* studies demonstrated that knockdown of MCT4 could inhibit the proliferation of bladder cancer cells. Furthermore, knockdown of MCT4 led to the significant increase of ROS and MDA levels in 5637 cells and ferroptosis in 5637 cells induced by ferroptosis inducers including RSL3 (APExBIO) and erastin (APExBIO) via inhibition of AMPK-related proteins. Moreover, knockdown of MCT4 inhibited autophagy in 5637 cells, while siMCT4 promoted inhibition of autophagy by CQ (an autophagy inhibitor), which increased the level of apoptosis.

**Conclusion:**

This study confirmed that knockdown of MCT4 could affect oxidative stress and induce ferroptosis and inhibition of autophagy, thus suggesting that MCT4 may be a potential target for the treatment of bladder cancer.

## 1. Introduction

Bladder cancer (BCa) is one of the most common malignant tumors in urology [1]. Adjuvant chemotherapy after surgery is the current mainstream therapy [2]. However, due to the high recurrence rate and drug resistance [3, 4], current therapies are not ideal for bladder cancer [5, 6]; thus, finding new drugs and therapies is vital for treating bladder cancer. Ferroptosis is a form of programmed cell death different from apoptosis and necrosis [7–9]. It is accompanied by highly concentrated mitochondria in cells, thickening of mitochondrial membrane, and disappearance of mitochondrial cristae [10].

MCT4 is a lactic acid transporter expressed on the cell membrane, which can excrete lactic acid produced by anaerobic glycolysis from the cell [11]. Knockout of MCT4 leads to the accumulation of intracellular lactic acid, causing excessive intracellular production of ROS [12]. Previous studies have confirmed that extracellular lactic acid is not only one of the main carbon sources for lipid synthesis but also can regulate the production of lipids via AMPK, signal transduction molecules, and STAT3 [13]. Besides, AMPK is also a key sensor of cellular energy [14]. Both ferroptosis and autophagy can be affected by the AMPK signaling pathway [15–17]. MCT4 is highly expressed in bladder cancer tissue and can affect the proliferation of bladder cancer cells [18]. MCT1, which is similar in function and structure to MCT4, can affect the AMPK pathway via the regulation of lactate to affect ferroptosis. However, the mechanism through which MCT4 affects bladder cancer cell growth and cell death remains unclear. Therefore, the aim of this study was to reveal the role of MCT4 so as to provide new targets for bladder cancer.

## 2. Materials and Methods

### 2.1. Cell Culture and Transfection

The human bladder cancer cells 5637 were purchased from the National Biomedical Experimental Cell Resource Bank in Beijing, China, and were cultured in RPMI-1640 with 10% FBS in a 5%CO_2_ humidified atmosphere at 37°C according to guidelines. 10% fetal bovine serum and RPMI-1640 were purchased from Biological Industries.

siRNAs used in the knockdown of the MCT4 group and the negative control group were synthesized by GenePharma (Suzhou, China) and transfected into 5637 cells using Lipofectamine 2000 (Thermo Fisher Scientific, USA) according to the manufacturer's protocol. The medium was replaced with fresh medium after 36 hours. Sequences of siRNAs were shown in Table 1.

### 2.2. Quantitative Reverse Transcription Polymerase Chain Reaction

Total cellular RNA was isolated using (Omega, Bio-Tek, USA). RNA (2 *μ*g) was reverse transcribed into cDNA using reverse transcriptase (TIANGEN Biotech, China). RT-PCR was performed in a 20 *μ*l reaction system containing 2× Talent qPCR PreMix (TIANGEN Biotech, Beijing, China) and specific primers according to the manufacturer's instructions. Primers were designed to amplify SLC16A3 and GAPDH. The sequences of these primer pairs are shown in Table 2.

### 2.3. Western Blot

Cells and tissues were collected and mixed with PMSF (Beyotime, China) containing protease inhibitor. The total protein was extracted using the ExKine™ Pro Animal Cell/Tissue Total Protein Extraction Kit (Abbkine, USA). BCA protein concentration assay kit (Beyotime, China) was used to determine total protein concentration. The extracted protein (20 *μ*g per well) was separated with 8-10% SDS-PAGE gel and transferred to the PVDF membrane (Merck Millipore; Billerica, MA, USA). The membranes were blocked with QuickBlock™ for 1 hour and incubated with primary antibodies overnight at 4°C. Then, membranes were incubated with secondary antibodies (dissolved in antibody dilution buffer (Beyotime, China) for 1 hour at room temperature and then assessed using chemiluminescence (Abbkine, California, USA).

Primary antibodies used in this study were MCT4 (proteintech, 22787-1-AP), Vinculin (proteintech, 26520-1-AP), SLC7A11/xCT (proteintech, 26864-1-AP), AIFM2/FSP1 (proteintech, 20886-1-AP), pAMPK*α* (cst, 50081), ACC (cst, 3676) and LC3A/B (cst, 12741) and Tubulin (proteintech, 11224-1-AP). The dilution ratios of the above primary antibodies were 1 : 1000. Secondary antibodies used in this study were HRP Goat Anti-Mouse IgG (H+L) (abclonal, AS003) and HRP Goat Anti-Rabbit IgG (H+L) (abclonal, AS014). The dilution ratios of the above secondary antibodies were 1 : 5000.

### 2.4. Lipid Peroxidation Assays

MDA Assay Kit (Dojindo, Japan) was used to evaluate the level of lipid peroxidation of the knockdown of the MCT4 group and the negative control group. The level of MDA was expressed as the ratio of absorbance values between treated cells and control cells. All results were obtained from three independent experiments performed in triplicate.

### 2.5. Lipid ROS Assays by LiperFluo

Twenty-four hours before the assay, the transfected 5637 cells (100,000 cells/well) were seeded in 6-well plates. Cells were treated with 2.5 *μ*mol/l of RSL3 (APExBIO) to induce ferroptosis. After 24 hours, the cells were incubated with the diluted LPO probe (10 *μ*Mol/ml, (Dojindo, Japan) for 40 min in the dark in a 37°C incubator, taken out and washed with PBS 3 times, and then imaged by a fluorescence microscope (Olymbus IX83, Japan). All results were obtained from three independent experiments performed in triplicate.

### 2.6. Transmission Electron Microscopy (TEM)

The 5637 cells treated with erastin (APExBIO) 2.5 *μ*mol were gently scraped with a spatula, and the cell pellet was collected after centrifugation at 800 r. The collected cells were fixed in 2.5% and 1% glutaraldehyde (Servicebio, China). After fixation with osmic acid (Ted Pella Inc., USA), cells were diluted with PBS (pH 7.4) for dehydration. The samples were embedded in Epon, cut using an ultramicrotome (LeicaUC7, German), and then stained with 2% uranyl acetate. Finally, the sections were analyzed by TEM (Hitachi HT7700, Japan).

### 2.7. Establishment of Stable Cell Line

For the production of lentiviruses, HEK293T cells were seeded in 100 mm plates. At 70-80% confluence, 293T cells were cotransfected with plasmid vectors of the target gene, packaging plasmids, and envelope plasmids at a ratio of 4 : 3 : 2 using Lipofectamine 2000 (Thermo Fisher Scientific, USA). Twelve hours after transfection, the medium was replaced with a fresh medium. Viral particles were collected 48 hours after transfection, filtered through a 0.45 *μ*m sterile filter, and concentrated by ultracentrifugation (4000 g, 20 min) using an ultracentrifuge (Beckman-Coulter XL-100K, German) at 4°C. Then, viral particles were resuspended with 2 ml fresh medium containing 9 *μ*M polybrene (Beyotime, China). Target cells were seeded in six-well plates and cultured in an incubator for 24 hours. Lentiviral-transduced cells were selected with a medium containing 10 *μ*g/ml puromycin (Beyotime, China) for 7 days, and the medium was replaced once a day.

### 2.8. Colony Formation Assay

The stably transfected cells of the knockdown of the MCT4 group and the negative control group were trypsinized, counted, and reseeded at 4000/well in 60 mm plates to form colonies. The medium was replaced every 2 days. After 14 days of culture, the colonies were fixed with 6% glutaraldehyde (Amresco) for 20 min and stained with a mixture of 0.5% crystal violet for 15 min. After staining, the samples were carefully rinsed with tap water to remove the crystal violet. Plates with colonies were dried in normal air at room temperature. All results were obtained from three independent experiments.

### 2.9. Caspase-3/7 Activity Assay

Transfected 5637 cells (average 1.5 × 10^4^ per well) were seeded in white 96-well plates. The cells were treated with CQ (Selleck) (1 *μ*M) for 24 h after cell adhesion for 15 hours. Caspase-3/7 activity was detected by a multiplate reader (Thermo Fisher Scientific, USA) using the Caspase-3/7 Fluorescence Detection Kit (Promega, USA) according to the manufacturer's instructions. All results were obtained from three independent experiments.

### 2.10. Assessment of Apoptosis

Transfected 5637 cells (6 × 10^5^) were cultured on 60 mm tissue culture plates and treated with CQ (1 *μ*M) (Selleck) for 24 hours. Then, cells were stained with the Annexin-V-FITC Apoptosis Kit (Dojindo, Japan) to analyze the apoptosis by flow cytometry (Thermo Fisher Scientific, USA) immediately. All results were obtained from two independent experiments performed in triplicate and expressed as mean ± SD.

### 2.11. Fluorescence Microscope

The effect of MCT4 on the autophagic flux of 5637 cells AdPlus-mCherry-GFP-LC3B (Beyotime, China) was used to detect the effect of MCT4 on the autophagic flux of 5637 cells. Cells in the logarithmic growth phase were seeded in 24-well plates (5 × 10^4^ per well) and cultured in a CO_2_ incubator (5% CO_2_, 37°C). Adenovirus transfection was performed when the cell confluency was 50%. Each plate of cells was infected with mCherryGFP-LC3B (Beyotime, China) adenovirus (MOI = 40) for 24 h. Cells were then cultured for another 24 h after replacing the adenovirus solution with a fresh medium and transfection was observed under an inverted fluorescence microscope (OLYMBUS IX83, Japan). The efficiency of transfection was assessed by an inverted fluorescence microscope (OLYMBUS IX83, Japan). The autophagic flux was evaluated by a fluorescence microscope (OLYMBUS IX83, Japan) after removing the medium containing the adenovirus solution.

To determine ROS levels in 5637 cells, DCFH-DA (10 mM) (Jiancheng Bio, China) was added to a serum-free medium to 10 *μ*M according to the manufacturer's instructions. The adherent cells after treatment were gently washed 3 times with PBS and were incubated with 10 *μ*M DCFH-DA in an incubator at 37°C for 40-50 minutes in the dark. The level of ROS of cells was evaluated with a fluorescence microscope (OLYMBUS IX83, Japan) after washing 3 times with PBS.

### 2.12. *In Vivo* Tumor Xenograft Experiments

All animal studies (including the mice euthanasia procedure) were done in compliance with the regulations and guidelines of Dalian Medical University institutional animal care and conducted according to the AAALAC and the IACUC guidelines (IACUC animal approval protocol #AEE21073). 5637 cells were stably transfected with SLC16A3-shRNA1 or NC-shRNA. Transfected 5637 cells (600 × 10^4^ cells/100 *μ*l) were then injected into the right axilla of 6-week-old male nude mice (*n* = 12; Charles River, Beijing, China). Tumor volume was measured every four days. Mice were sacrificed 65 days after injection, after which volume and weight of tumors were recorded.

### 2.13. Statistical Analysis

All data were statistically analyzed using GraphPad Prism 7.0 (GraphPad 8.0, Inc., San Diego, CA, USA). Measurement data were expressed as mean ± standard deviation (mean ± SD). The comparison between the knockdown of the MCT4 group and the negative control group was analyzed by the Student *t*-test, and the comparison between multiple groups was analyzed by one-way ANOVA. A *p* value < 0.05 was considered statistically significant (^∗^*p* < 0.05, ^∗∗^*p* < 0.01, ^∗∗∗^*p* < 0.001, and ^∗∗∗∗^*p* < 0.0001).

## 3. Results

### 3.1. Knockdown of MCT4 significantly inhibits bladder carcinoma proliferation in vivo and in vitro

**Figure 1:**
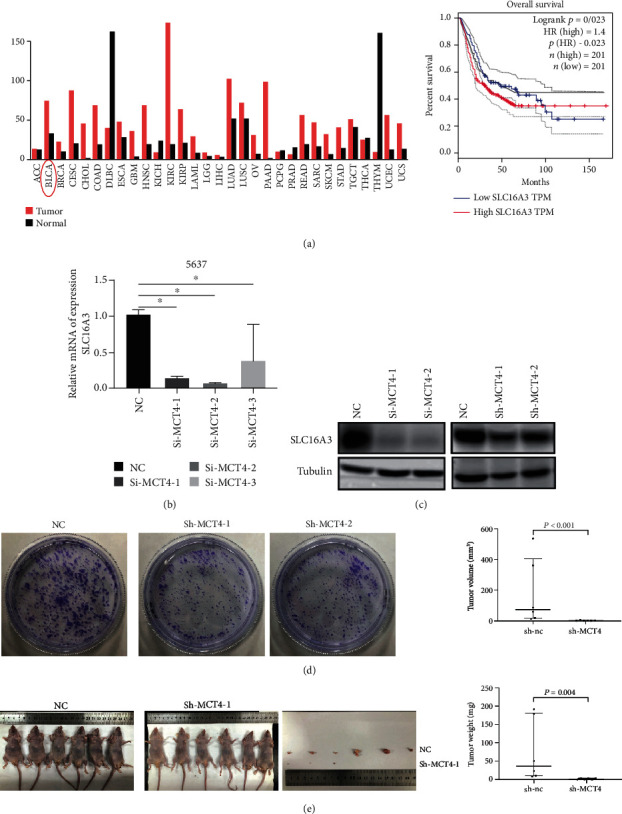
MCT4 was positively associated with poor prognosis in bladder cancer patients. Among 402 bladder cancer patients, the survival rate of 201 patients with high expression of MCT4 was significantly lower than that of 201 patients with low expression of MCT4. *p*(HR) was 0.023, and HR was 1.4. (b) The knockdown efficiency of MCT4 mRNA was detected by qRT-PCR. GAPDH was used as an internal reference. (c) Western Blot assay was used to detect the knockdown efficiency of MCT4. Tubulin was used as an internal control. (d) Clone formation assay was performed to detect the clone formation ability of bladder cancer cells after knockdown of MCT4. (e) Subcutaneous tumorigenicity of 5637 cells in nude mice after knockdown of MCT4.

The genes related to oxygen metabolism were screened out by bioinformatics in the previous studies, and the Kaplan-Meier method was used to detect the correlation between each gene and the survival rate of bladder cancer patients. Thus, MCT4 was selected, and the GEPIA database revealed 402 bladder cancer patients. Among them, there were 201 patients with high expression of MCT4 and poor prognosis (Figure 1(a)) and 201 patients with high expression of MCT4 and poor prognosis (Figure 1(a)). Besides, it has been reported that MCT4 is associated with poor prognosis in bladder cancer patients [19].

Subsequently, we established a knockdown model of 5637 cell lines by RNAi and lentiviral to detect the effect of knockdown of MCT4 on bladder cancer cells. The knockdown efficiency was verified by qPCR and Western Blot assays, which showed that the expression of MCT4 in the knockdown group was significantly reduced (Figure 1(b) and 1(c)). In order to verify that MCT4 is related to the proliferation in 5637 cells, we performed a clone formation assay, finding that knockdown of MCT4 significantly inhibited the proliferation of 5637 cells. This suggested that the knockdown of MCT4 significantly inhibits the proliferation of 5637 cells *in vitro* (Figure 1(d)). Besides, the tumorigenic ability of 5637 cells in nude mice was significantly decreased, and the volume and weight of tumors in the knockdown group were significantly lower than those in the control group, which suggested that the knockdown of MCT4 significantly inhibits the proliferative capacity of 5637 cells (Figure 1(e)).

### 3.2. Knockdown of MCT4 induces ferroptosis in bladder cancer cells

**Figure 2:**
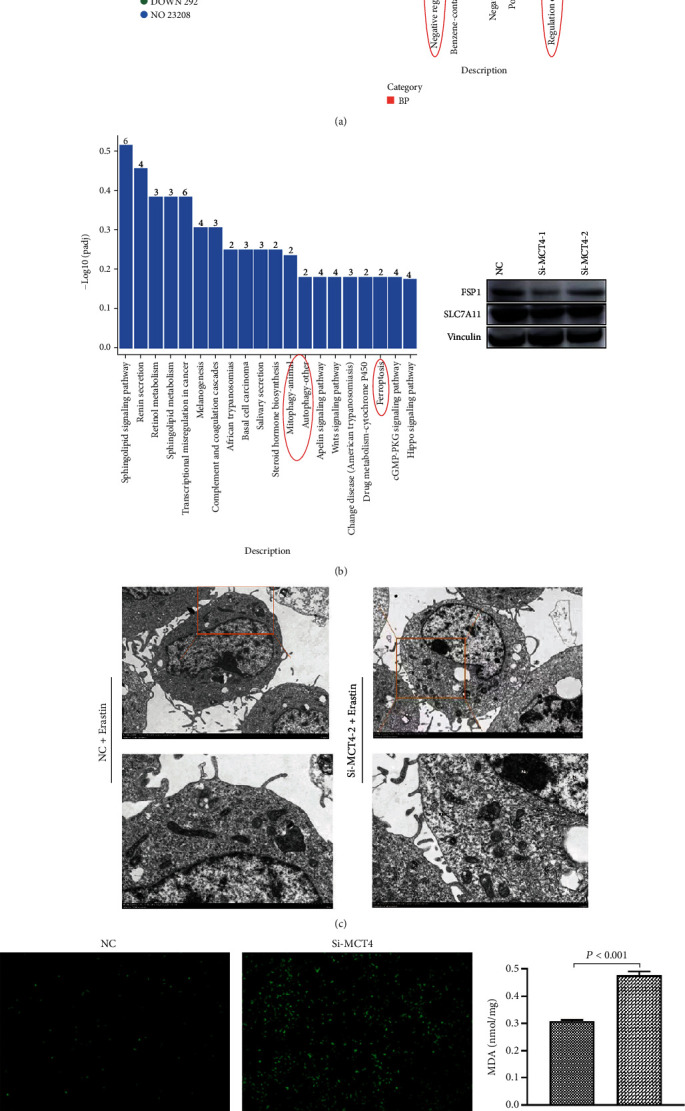
Bladder cancer cells underwent ferroptosis after the knockdown of MCT4. (a) Pathway analysis showed that ferroptosis and autophagy-related pathways were activated after the knockdown of MCT4. (b) Western Blot assay detected the expression levels of FSP1 and SLC7A11 proteins. (c) The intracellular morphology was evaluated by TEM. (d) DCFH-DA probe detected ROS in 5637 cells in knockdown of the MCT4 group and control group after induction with 2.5 *μ*M RSL3 (APExBIO) for 24 h. Scale bar = 200 *μ*m. (e) MDA assay detected the changes of lipid metabolism disorder in knockdown of the MCT4 group and control group after 24 h induction with 2.5 *μ*M RSL3 (APExBIO). (f) LPO assay detected the level of lipid peroxidation in 5637 cells after knockdown of MCT4, and fer-1 (APExBIO) was a specific inhibitor of ferroptosis. Scale bar = 50 *μ*m. ^∗^*p* < 0.05, ^∗∗^*p* < 0.01, and ^∗∗∗∗^*p* < 0.001.

The gene expression profile after knockdown of MCT4 was achieved via RNA-seq, where 182 genes were upregulated and 292 genes were downregulated in the volcano plot (Figure 2(a)). Cluster analysis showed that a large number of signaling pathways were activated after knockdown, and KEGG enrichment analysis showed that oxygen metabolism, ferroptosis, and autophagy pathways were mainly enriched (Figure 2(a)). It has been reported that MCT4 mainly affects intracellular ROS levels and oxidative stress [12, 18]. Besides, combined with the above sequencing results, it was speculated that MCT4 affected cell ferroptosis by regulating oxidative stress. Therefore, the expression of ferroptosis protein markers, including FSP1and SLC7A11 after the knockdown of MCT4in 5637cell lines was determined to verify the relationship between MCT4 and ferroptosis. The results showed that the expressions of FSP1 and SLC7A11 in 5637 cells were significantly downregulated after knockdown, thus suggesting that the cells underwent ferroptosis (Figure 2(b)). Moreover, TEM revealed that after knockdown of MCT4, the mitochondria of 5637 cells shrank, were rounded, and vacuolized following erastin treatment (APExBIO) (Figure 2(c)). DCFH-DA revealed that the level of ROS in the knockdown of the MCT4 group increased after treatment with RSL3 (APExBIO) (Figure 2(d)), while dialdehyde (MDA) level was significantly elevated (Figure 2(e)).

To further verify the relationship between MCT4 and ferroptosis, it was confirmed that the level of lipid ROS in the knockdown of the MCT4 group was significantly increased after treatment of RSL3 (APExBIO) by LPO assay, which could be rescued by fer-1 (APExBIO), the ferroptosis-specific inhibitor (Figure 2(f)). These results showed that knockdown of MCT4 aggravates ferroptosis in 5637 cells.

### 3.3. MCT4 Regulates Ferroptosis by Controling the Expression Levels of Key Proteins in the AMPK Pathway

**Figure 3:**
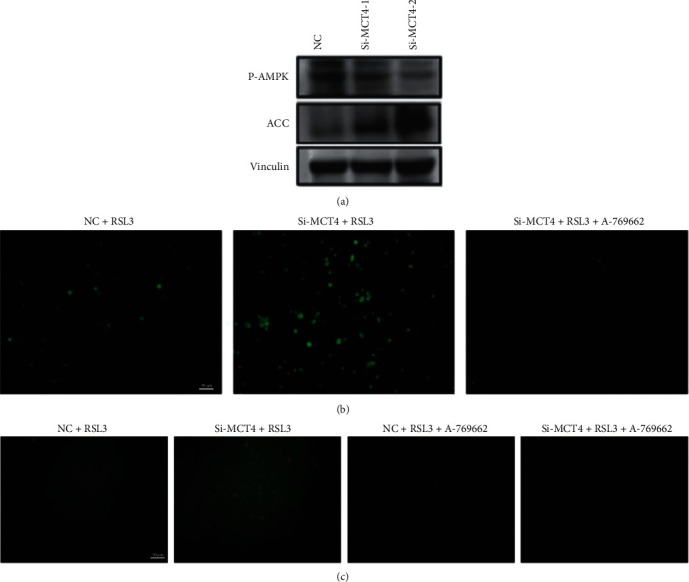
AMPK pathway was activated after the knockdown of MCT4. (a) Western Blot assay was performed to detect the expression of P-AMPK and ACC. (b) Fluorescence microscopy (OLYMBUS IX83, Japan) was used to detect the level of intracellular ROS in knockdown of the MCT4 group treated with A-769662 (Selleck) and 2.5 *μ*M RSL3 (APExBIO) simultaneously for 24 h. Scale bar = 50 *μ*m. (c) Fluorescence microscopy (OLYMBUS IX83, Japan) was used to detect the level of lipid ROS in knockdown of the MCT4 group and control group after simultaneous treatment with A-769662 (Selleck) and 2.5 *μ*M RSL3 (APExBIO) for 24 h, scale bar = 50 *μ*m.

Previous studies have reported that ferroptosis and autophagy are affected by the AMPK pathway [15, 20]. In this study, the expression of key proteins in the AMPK pathway was detected by Western Blot. The results showed that expressions of p-AMPK decreased and expression of ACC increased in 5637 cells with knockdown of MCT4, which suggested the inhibition of the AMPK pathway (Figure 3(a)). Therefore, AMPK agonist A-769662 was used to rescue the ferroptosis induced by siMCT4, revealing that AMPK agonist A-769662 could rescue ferroptosis induced by siMCT4. These results suggest that the knockdown of MCT4 regulates ferroptosis by regulating the expression levels of key proteins in the AMPK pathway. These results further showed that the AMPK agonist A-769662 significantly inhibits the elevated ROS levels caused by knockdown of MCT4 (Figure 3(b)) and the level of lipid ROS in the cells with knockdown of MCT4 (Figure 3(c)) in 5637 cells.

### 3.4. Knockdown of MCT4 inhibits the Autophagy in Bladder Cancer Cells

**Figure 4:**
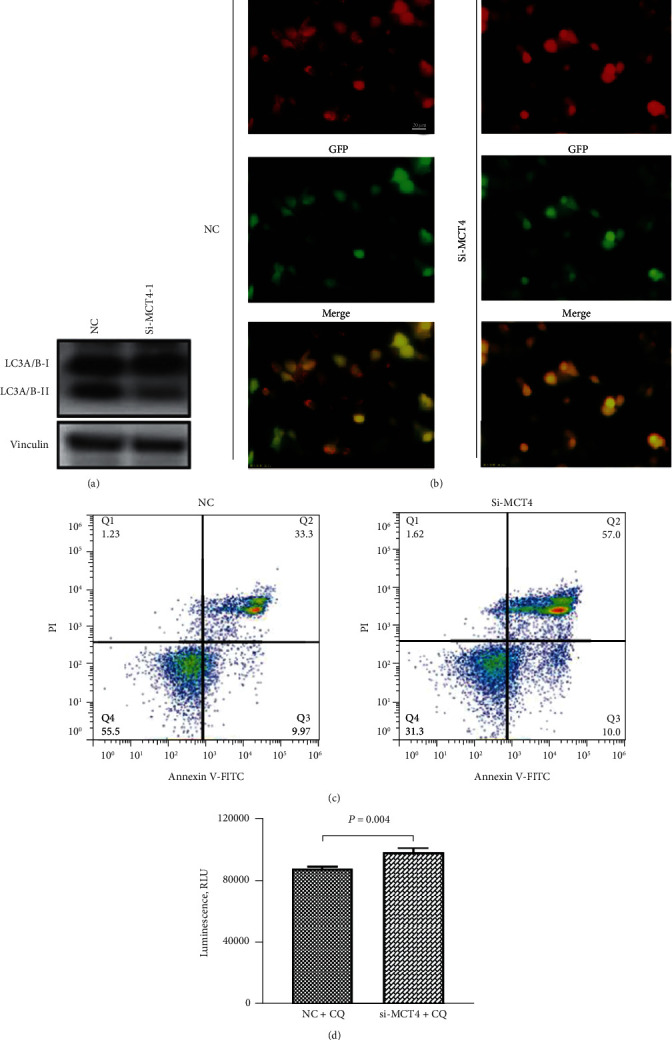
Autophagy in bladder cancer cells was inhibited after knockdown of MCT4. (a) Western Blot assay was used to detect the changes in the protein expression levels of autophagy-related proteins, including LC3A/B I and LC3A/B II in 5637 cells with MCT4 knockdown and the control group. Vinculin was used as an internal control. (b) Fluorescence microscopy (OLYMBUS IX83, Japan) detected that autophagy was inhibited in 5637 cells after transient transfection of siMCT4 for 48 h by AdPlus-mCherry-GFP-LC3B. Scale bar = 20 *μ*m. (c) Flow cytometry detected the changes of apoptosis in the knockdown group and the control group after treatment of 1 *μ*M CQ (Selleck) for 24 h. (d) Changes of caspase-Glo3/7 in the knockdown of the MCT4 group and the control group after treatment of 1 *μ*M CQ for 24 h were detected by a fluorescence microplate reader. ^∗∗^*p* = 0.004.

In addition, we also tested whether the knockdown of MCT4 could affect autophagy. RNA-seq showed that the autophagy pathway was enriched after the knockdown of MCT4 by KEGG enrichment analysis. In this study, the expression of autophagy-related proteins was detected by Western Blot. The results showed that the expression of LC3A/B-II was decreased after the knockdown of MCT4, indicating that autophagy was significantly inhibited after the knockdown of MCT4 (Figure 4(a)) in 5637 cells. It was also found that the red puncta were significantly reduced after knockdown of MCT4 through the AdPlus-mCherry-GFP-LC3B reporter system assay, which further indicated that knockdown of MCT4 inhibited the autophagy (Figure 4(b)). In order to verify the relationship between inhibition of autophagy induced by knockdown of MCT4 and apoptosis in 5637 cells, we continued to apply CQ (Selleck) to induce autophagy inhibition in cells. After AVPI staining, it was shown that the knockdown of MCT4 could significantly promote cell apoptosis detected by flow cytometry (Figure 4(c)). Fluorescence microplate reader showed that the luminescence intensity of caspase-3/7 in knockdown of the MCT4 group was higher than that in the control group after treatment of CQ (Selleck) (Figure 4(d)).

## 4. Discussion

In the present study, we demonstrated that the knockdown of SLC16A3 could damage cells by promoting ferroptosis in 5637 cells. SLC16A3 is a gene located on the long arm of chromosome 17, encoding the MCT4 [21]. MCT4 is an important metabolic target in tumor biology that transports monocarboxylic acids through proton coupling. The most affected are lactic acid, ketone bodies, and pyruvate [21]. It has been reported that inhibition of MCT4 can induce apoptosis to damage liver cancer cells [22]. Our results showed that in 5637 cells, knockdown of MCT4 significantly inhibited the clonogenic ability and proliferation ability of tumor cells. Meanwhile, GEPIA database analysis showed that the long-term survival of bladder cancer patients with high expression of MCT4 was significantly lower than that of bladder cancer patients with low expression of MCT4, which suggested that MCT4 may be a potential target for bladder cancer therapy.

MCT4 can regulate intracellular pH balance and content of lactic acid by mediating intracellular and extracellular lactic acid transport, thereby regulating oxidative stress in tumor cells [11]. Our results showed that the oxidative stress in 5637 cells significantly increased, and a large amount of ROS was produced after the knockdown of MCT4. The RNA-seq analysis of the knockdown of the MCT4 group and the control group at the same time showed that a large number of ferroptosis-related genes were significantly changed after the knockdown of MCT4. Moreover, the expressions of ferroptosis-related protein markers, including FSP1 and SLC7A11, were detected in cells with MCT4 knockdown and in the control group; yet, the expressions of FSP1 and SLC7A11 in the MCT4 knockdown group were lower than in the control group.

In order to further study the relationship between MCT4 and ferroptosis, MDA and LPO assays were performed in knockdown of the MCT4 group and control group in 5637 cells. The morphological changes of cells with knockdown of MCT4 were determined by TEM. The results showed that the level of lipid metabolism disorder and lipid peroxidation increased in the MCT4 knockdown group. Besides, the mitochondrial condensation, mitochondrial membrane thickening, and disappearance of mitochondrial ridge occurred in the MCT4 knockdown group, showing typical morphological changes of ferroptosis, which could be rescued by fer-1 (APExBIO), a specific inhibitor of ferroptosis. These results showed that knockdown of MCT4 induces ferroptosis in 5637 cells. In addition, Western Blot data showed that the AMPK pathway was significantly inhibited after knockdown with downregulation of the phosphorylation of AMPK and upregulation of ACC. Agonist of the AMPK pathway could inhibit the elevated lipid peroxidation level by knockdown of MCT4. It was found that the knockdown of MCT4 induces ferroptosis by inhibiting the AMPK pathway in 5637 cells.

RNA-seq and Western Blot proved that MCT4 could regulate ferroptosis through the AMPK pathway. Under various physiological and pathological conditions, AMPK can be activated after phosphorylation by upstream kinases. Activated AMPK can regulate various metabolic processes, including autophagy and phosphorylate autophagy-related proteins of mTORC1-ULK1-PIK3C3/VPS34 complex to directly promote autophagy [23, 24] or indirectly promote autophagy by regulating transcription factors including FOXO3, TFEB, and BRD4 to regulate downstream autophagy-related genes [25, 26]. Moreover, the RNA-seq was also enriched in autophagy. Thus, further studies were performed in order to establish whether the knockdown of the gene also affected autophagy in 5637 cells.

The results showed a significant autophagy inhibition after the knockdown of MCT4 in 5637 cells. Changes in autophagy-related proteins showed that the knockdown of the MCT4 gene significantly inhibited autophagy in 5637 cells. In order to verify the changes in autophagy after knockdown of MCT4, fluorescence microscopy (OLYMBUS IX83, Japan) was used to perform experiments and observe autophagy-related proteins LC3A/B I and LC3A/B II changes, thereby judging the occurrence of autophagy. In order to further understand the effect of gene knockdown on autophagy inhibition, we applied the autophagy inhibitor CQ (Selleck) to amplify the inhibitory effect of autophagy and detect the effect of MCT4 on the degree of apoptosis by Annexin-V-FITC Apoptosis Kit. After adding CQ (Selleck) to the MCT4 cell line, the degree of apoptosis increased, thus indicating that knockdown of MCT4 can induce the inhibition of autophagy, thereby promoting cell apoptosis. Numerous reports have shown that the inhibition of autophagy and ferroptosis of cells is an antagonistic relationship [27]. AMPK is a protein kinase called adenylate-activated protein kinase, which is an energy sensor in cells. AMPK is responsible for controlling and regulating the energy supply system of cells, which at the same time responds to the environment, improving the adaptability of cells themselves [28]. Inhibition of AMPK has been reported to inhibit autophagy, and activation of AMPK has also been reported to inhibit ferroptosis [15, 29], which is consistent with the results of our experiment, where the knockdown of the gene affected the AMPK pathway, thereby inducing ferroptosis and inhibiting autophagy. This may be due to the following two reasons, inhibited protective autophagy in this study induced ferroptosis in cells, or the abnormal lactate metabolism caused by the knockdown of MCT4 caused the disorder of the autophagy process and the ferroptosis process, respectively. At this time, the cell death process depends on the type of inducer added. If it is an inducer of ferroptosis, then the knockdown of MCT4 can induce ferroptosis, and if it is an autophagy inhibitor, the knockdown of MCT4 can increase the degree to which cells are inhibited by autophagy, thereby increasing apoptosis.

Regarding the inhibition of autophagy caused by siMCT4in 5637 cells, although no study has reported that siMCT4 can cause inhibition of autophagy, it has been reported that tumor hypoxia and acidosis are the main features of the bladder tumor microenvironment (TME) [30, 31]. Cells can activate autophagy in an acidic, hypoxic environment, thereby promoting the proliferation of bladder cancer cells. This study speculated that siMCT4 inhibited the proliferation of bladder cancer cells by changing the acidic microenvironment and inhibiting autophagy.

To sum up, our results revealed that siMCT4 could induce ferroptosis in 5637 cells and act as a sensitizer to enhance ferroptosis inducers. Besides, siMCT4 could also induce inhibition of autophagy in 5637 cells and enhance tumor cell apoptosis reduced by autophagy inhibitor CQ (Selleck).

## Figures and Tables

**Table 1:** Sequences of siSLC16A3.

Names of siRNAs	Sequences
siRNA in the negative control group (NC) forward	5′-UUCUCCGAACGUGUCACGUTT-3′
siRNA in the negative control group (NC) reverse	5′-ACGUGACACGUUCGGAGAATT-3′
SLC16A3 siRNA1 forward	5′-GGAGCAUCAUCCAGGUCUATT-3′
SLC16A3 siRNA1 reverse	5′-UAGACCUGGAUGAUGCUCCTT-3′
SLC16A3 siRNA2 forward	5′-GCAUUAGGAAGAAGCCCAATT-3′
SLC16A3 siRNA2 reverse	5′-UUGGGCUUCUUCCUAAUGCTT-3′

**Table 2:** Primer sequences of SLC16A3, GAPDH.

Names of primers	Sequences
SLC16a3 forward primer	5′-AGGTCCGCTCTGCAGTGTGT-3′
SLC16a3 reverse primers	5′-AAACCCAACCCCGTGATGA-3′
GAPDH forward primer	5′-GCTGAGAACGGGAAGCTTGT-3′
GAPDH reverse primers	5′-GCCAGGGGTGCTAAGCAGTT-3′

## Data Availability

The datasets used and/or analyzed during the current study are available from the corresponding author on reasonable request.
